# A Case of Biliary Pancreatitis Which Subsided after Endoscopic Sphincterotomy during Pregnancy

**DOI:** 10.5402/2011/481980

**Published:** 2011-04-11

**Authors:** Gulbanu Erkan, Ibrahim Dogan, Seren Ozenirler, Candan Tuncer

**Affiliations:** ^1^Division of Gastroenterology, Department of Internal Medicine, Faculty of Medicine, Ufuk University Hospital, Mevlana Bulvarı no. 86–88, Balgat, 06520 Ankara, Turkey; ^2^Division of Gastroenterology, Department of Internal Medicine, Faculty of Medicine, Gazi University Hospital, 06500 Ankara, Turkey

## Abstract

A 24-year-old pregnant patient was referred to us because of pain and tenderness in the right upper quadrant. Her liver enzymes and bilirubin levels were elevated; an abdominal ultrasound examination revealed gallstones within the gallbladder. Diagnosis of biliary pancreatitis was established based on elevated amylase levels. Oral intake was withheld; intravenous antibiotic therapy and total parenteral nutrition were administered. An endoscopic sphincterotomy without the use of fluoroscopy was performed. Abdominal pain and elevated serum amylase levels subsided after this procedure. In our case, biliary pancreatitis, which developed during pregnancy, responded well to the endoscopic sphincterotomy, and this procedure obviated the need for surgical intervention and prevented the recurrence of pancreatitis.

## 1. Introduction

Cholelithiasis and pancreatitis are two commonly encountered entities during pregnancy. In the Framingham cohort, the incidence of cholelithiasis shows a positive correlation with the number of pregnancies, especially after the fourth pregnancy [1]. 

The increase in the lithogenicity of the bile and decrease in the motility of the gallbladder causes the increase in the incidence of cholelithiasis during pregnancy [2].

With conservative measures only, the risk of recurrence of biliary pancreatitis during pregnancy is 72% percent [3]. Thus, the importance of preventive interventions aiming to reduce the risk of recurrence of pancreatitis during pregnancy cannot be overemphasized. 

In this context, we are presenting a case of acute biliary pancreatitis during pregnancy who responded well to endoscopic sphincterotomy.

## 2. Case Report

A 24-year-old female patient in the 32nd week of her second pregnancy was referred to the Department of Gastroenterology, because of pain in the right upper quadrant that radiated to the back and the epigastrium. On palpation, the patient had displayed tenderness in the right upper quadrant. Sclerae were icteric. An abdominal ultrasound examination revealed homogenous liver parenchyma, and a few gallstones were visualized in the gallbladder, the largest measuring 23 mm in diameter. The transverse diameter, wall thickness of the gallbladder, and intrahepatic and extrahepatic bile ducts were normal. A biochemical analysis of the serum revealed an elevated serum amylase level (1724 IU/mL); liver function tests and serum bilirubin levels were as follows: aspartate aminotransferase—287 IU/mL, alanine aminotransferase—319 IU/mL, alkaline phosphatase—261 IU/mL, gamma gluthamil transferase—83 IU/mL, total bilirubin—2,87 mg/dL, and direct bilirubin—2,44 mg/dL. Laboratory findings on admission and through followup are summarized in Table 1. The patient's serum triglyceride and calcium levels were normal. The patient was diagnosed as having acute biliary pancreatitis. As a result, oral intake was withheld. Intravenous fluid therapy, Ampicillin + Sulbactame 3 × 1 g/day, and total parenteral nutrition were administered. On the second day of hospitalization, an endoscopic sphincterotomy was performed without the use of fluoroscopy, and the common bile duct was selectively cannulated with a guidewire using direct video endoscopic control. A catheter was advanced over the guidewire into the bile duct. Bile was aspirated into the catheter to confirm cannulation of the bile duct, and the sphincterotomy was performed. The patient's abdominal pain and the elevated amylase and lipase levels subsided after this procedure. Uterine contractions, which began on the fifth day of the hospitalization, could not be halted with magnesium infusion, and the patient gave birth to a premature but otherwise healthy infant with a Caesarian section in the 32nd gestational week.

## 3. Discussion

The incidence of cholelithiasis is increased in pregnancy, especially in young multiparous women [1]. On the other hand, the incidence of pancreatitis during pregnancy is not increased, but it is more common in the third trimester. The most common cause of pancreatitis during pregnancy is cholelithiasis [2]. 

In a series reported by Ramin et al., the prevalence of acute pancreatitis in pregnancy is one in 3333 pregnancies. In this series, the mean maternal age was 24, and 72% of patients were multiparous. Acute pancreatitis, due to biliary disease, developed in 68% of patients. The authors concluded that acute pancreatitis was generally associated with cholelithiasis in pregnant women, and the maternal prognosis was especially good with early supportive therapy and surgical therapy, if it was indicated [4]. 

Endoscopic sphincterotomy can be performed without general anesthesia or an abdominal incision. Baillie et al. reported a successful prophylactic endoscopic sphincterotomy in a single pregnant patient who had developed gallstone pancreatitis [5]. 

Barthel et al. also reported that, in pregnancy-associated gallstone pancreatitis, endoscopic sphincterotomy prevents recurrence of pancreatitis and the need for a cholecystectomy during gestation [6].

In our case, an abdominal ultrasound examination was performed in order to clarify the etiology of right upper quadrant pain that developed in the 32nd week of pregnancy. As a result, cholecystolithiasis was discovered. After the diagnosis of biliary pancreatitis had been established with clinical and biochemical evidence, an endoscopic sphincterotomy was performed without the use of fluoroscopy, and the clinical symptoms of pancreatitis subsided. There was no procedure related to maternal complication, but the delivery was preterm. Nevertheless, fetal development was consistent with gestational age, and no severe growth retardation or any other complications were encountered. 

In conclusion, the endoscopic sphincterotomy, which was performed for biliary pancreatitis during pregnancy, was efficacious and obviated the need for urgent surgical intervention and prevented the recurrence of the pancreatitis without causing any serious maternal or fetal complications.

## 4. Key Points

The incidence of cholelithiasis is increased in pregnancy, especially in young multiparous women.

The most common cause of pancreatitis during pregnancy is cholelithiasis.

With conservative measures only, the risk of recurrence of biliary pancreatitis during pregnancy is 72% percent.

Preventive interventions aiming to reduce the risk of recurrence of pancreatitis during pregnancy are beneficial.

## Figures and Tables

**Table 1:** Laboratory findings in the patient with biliary pancreatitis.

	Initial	Day 1	Day 4	Normal ranges
Serum amilase	1724	605	118	0–65 IU/mL
Total bilirubin	2.87	2.1	1.2	0.2–1.3 mg/dL
Direct bilirubin	2.44	1.8	0.5	0–0.5 mg/dL
ALT	319	197	163	23 IU/mL
AST	287	160	104	23 IU/mL
